# Impulsive and compulsive reading comprehension in the prison population

**DOI:** 10.1186/s12888-023-05372-w

**Published:** 2024-01-12

**Authors:** Lucas Muñoz-López, Francisca Serrano, María del Carmen López-Torrecillas, María Blasa Sánchez-Barrera, Ignacio Martín, Francisca López-Torrecillas

**Affiliations:** 1 Departamento de Personalidad, Evaluación y Tratamiento Psicológico, Facultad de Psicología, University of Granada, Campus de Cartuja S/N, 18071 Granada, Spain; 2 Centro de Investigación Mente, Cerebro y Comportamiento, University of Granada, Granada, Spain; 3 Consejería de Empleo, Empresa y Trabajo Autónomo de La Junta de Andalucía, Seville, Spain; 4 Departamento de Metodología de Las Ciencias del Comportamiento. Facultad de Psicología, University of Granada, Granada, Spain

**Keywords:** Reading, PROLEC-SE-R, Prisoners, Compulsivity, Impulsivity

## Abstract

**Background:**

Developmental dyslexia is characterized by reading and writing deficits that persist into adulthood. Dyslexia is strongly associated with academic underachievement, as well as impulsive, compulsive, and criminal behaviors.

The aims of this study were to investigate impulsive or compulsive reading comprehension, analyzing the differences in reading errors between two distinct groups —one with Antisocial Personality Disorder (ASPD) and another with Obsessive–Compulsive Personality Disorder (OCPD) and examine their correlation with criminal behavior within a prison population.

**Methods:**

We gathered data from 194 participants: 81 with ASPD and 113 with OCPD from a prison center. Participants took part in interviews to gather data on demographic, criminal, and behavioral data. Additionally, the participants underwent various assessments, including the International Examination for Personality Disorders; Symptom Inventory, and Battery for the Assessment of Reading Processes in Secondary and High School – Revised.

**Results:**

Our analysis revealed differences in reading skills between the ASPD and OCPD groups. Specifically, the OCPD group showed poorer performance on lexical selection, semantic categorization, grammar structures, grammatical judgements, and expository comprehension when compared with the ASPD group. Conversely, the OCPD group obtained higher scores on narrative comprehension relative to the ASPD group.

**Conclusions:**

The OCPD group showed slow lexical-phonological coding and phonological activation.

## Introduction

Language is essential for communication, and within confined communities such as prisons, it is an essential tool for conflict resolution. Various authors [1, 2] have demonstrated the high prevalence of language and communication disorders in the prison population. Language plays a crucial role in facilitating interactions with others, managing one’s emotions and actions, and promoting academic functioning. Consequently, individuals with oral language problems are susceptible to developing maladaptive and aggressive behaviors. Moreover, reading and writing skills are closely related to language ability, with poor language skills representing a known risk factor for dyslexia. Therefore, fostering the development of fundamental reading and writing skills among inmates is important, as these skills essentially form the foundation of their education.

In the prison population, different authors [1–11] have pointed out that between 5 and 90% are estimated to have reading and writing disorders. This considerable diversity in the prevalence of reading and writing difficulties is due to the lack of consensus regarding the definition of dyslexia, variations in testing methods — ranging from individual assessments to self-reports — and the heterogeneity of the analyzed samples. Thus, it is of clinical importance to establish a universally accepted international standard for evaluating language [11].

Reading deficits constitute one of the main characteristics of developmental dyslexia, and these problems persist into adulthood [12]. In the latter study, the authors analyzed whether adults with dyslexia showed reading disorders. Forty participants completed the battery of reading processes (PROLEC-SE; [13]) and the participants with dyslexia completed a self-reported test of reading problems. They found that the dyslexia group obtained significantly lower scores on all dimensions of the PROLEC battery (Lexical selection, Semantic categorization, Grammatical structures, Grammaticality judgments, Expository comprehension, Narrative comprehension, Word reading, Pseudoword reading, Grammatical structures, Punctuation marks, Pure reading comprehension, Mnemonic reading comprehension, and Oral comprehension), compared with the control group. It was concluded that the reading processes are slower and ineffective in individuals with dyslexia.

In addition, one study [14] has demonstrated that dyslexia is a major obstacle to learning, with various factors impacting reading abilities. The findings revealed that, in terms of semantic categorization, both children with dyslexia and younger participants in the control group obtained worse scores than the older children in the control group. In summary, the authors suggested that children with dyslexia struggle to benefit from the learning process, primarily due to a diminished coding efficiency. Consequently, these learning challenges contribute to academic underachievement, which is strongly correlated with the tendency to engage in delinquent behaviors [14].

Whilst there are limited studies [11–13, 15] that have directly evaluated reading skills in the adult prison population, it is well-established that factors such as school dropout, low educational level, and school failure serve as predictive indicators of criminal behavior. One study examined the relationship between efficacy beliefs, reading, and spelling skills (actual skills) in a sample of 600 males incarcerated in Norwegian prisons [15]. This study revealed a generally low reading and spelling ability, particularly in the youngest participants and those serving the longest sentences. It was concluded that the youngest inmates with a diagnosis of dyslexia and those facing sentences exceeding five years would benefit from a personalized education plan tailored to address their specific employment-related needs.

However, it is worth noting that dyslexia is not solely predicted by poor phonological ability. Sociocultural, educational, and emotional challenges are highly common factors in the development of both dyslexia and criminal behaviors [10, 16].

Criminal behaviors are strongly associated with personality and behavioral disorders [17]. In particular, impulsivity and compulsivity are traits that underlie violent behaviors [18]. The American Psychiatric Association [19] defines impulsivity as the execution of unplanned and rapid actions carried out without considering the possible negative consequences. Compulsivity has been defined as the appearance of recurrent behaviors whose goal is to reduce or avoid anxiety or distress [19]. Furthermore, compulsive maladaptive behavior has been classified as something planned, conscious, and never as a spontaneous act [20].

Dyslexia can be described as a neurodevelopmental disorder stemming from a biological predisposition toward showing poor phonological-processing skills, which hinder word decoding and the ability to recognize single words [21]. In this study, we make use of the well-documented finding that dyslexia and compulsivity share common underlying biological bases. Compulsive behavior is associated with increased frontal lobe activity, whereas impulsive behavior is associated with reduced frontal lobe activity [22]. Increased frontal lobe activity can characterize compulsive disorders, such as obsessive–compulsive disorder (OCD). Conversely, decreased frontal lobe activity can characterize impulsive disorders, such as substance use and antisocial personality disorder (SUD and APT). Numerous studies [23, 24] provide evidence to support the association between impulsivity and both substance use disorders (SUD) and violent or aggressive behavior. In contrast, research on compulsivity has been relatively limited. To date, compulsivity has only been examined in the context of OCD [20] and relatively few studies have analyzed its role in relation to addictive behaviors [25].

The biological bases of compulsivity share common brain areas and neural circuits with those related to language and communication. Numerous studies [26, 27] examining the biological bases of compulsivity have identified five main brain structures: 1) the dorsolateral prefrontal cortex, lateral orbitofrontal cortex, and the caudate nucleus, which collectively form the reverse learning circuit; 2) the supplementary motor area, premotor cortex and putamen, which constitute the habit learning circuit; 3) the cortical-striatum-thalamic-cortical circuits; 4) the fronto-limbic connections, and 5) the anterior cingulate nucleus. These areas have been extensively studied and linked to a wide range of language functions. More recent studies [28, 29] have found a strong association between each of the structures described above and various language functions.

The arcuate and superior longitudinal fascicles form the dorsal, sub-lexical, or phonological language pathway. Conversely, the uncinate, inferior longitudinal, and fronto-occipital fascicles constitute the ventral, lexical, or semantic language pathway. Both routes are specialized in interconnecting distant language areas, creating networks responsible for phonological recognition and language interpretation [12, 14, 28, 30].

Studies have been conducted to investigate lesions or abnormalities in four fascicles: [1) the arcuate fascicle, 2) the uncinate fascicle, 3) the longitudinal fascicle, and 4) the fronto-occipital fascicle]. These fascicles are interconnected with areas associated with compulsivity, and when affected, alterations in language can emerge, such as difficulties on tasks involving phonological and word repetition, verbal short-term memory, articulation, and the ability to distinguish between different phonemes [28]. The arcuate fascicle is composed of neuronal fibers that connect frontal, temporal, and parietal areas [31]. The uncinate fascicle extends from the lower frontal lobe, the fronto-orbicular area, to the temporal lobe, sending fibers to subcortical structures such as the hippocampus and the amygdala. The longitudinal fascicle can be separated into the superior, medium, and inferior segments. The inferior segment arises from the frontal and prefrontal lobe to connect with the occipital lobe, releasing fibers directly towards the posterior region of the parietal and temporal areas of the paleocortex and the insula. The superior segment connects the temporal area with the motor and premotor areas, passing through the angular and supramarginal gyrus [28]. The fronto-occipital fascicle shares the same pathway as the inferior longitudinal fascicle [32].

In light of the practical significance of understanding how reading abilities within the prison population may impact criminal behavior; the aim of this study was to investigate impulsive and compulsive reading comprehension in this population (assessed using the PROLEC-SE-R). Given the lack of research exploring reading processes in the context of OCPD and ASPD, the present study aimed to address this gap. In particular, the main reasons for conducting this study are as follows: First, the prevalence rates of Antisocial Personality Disorder (ASPD) in male prisoners range between 30 to 60%, while Obsessive Compulsive Personality Disorders (OCPD) affect over 63% of individuals in this population [33–35]. Second, individuals with ASPD and OCPD could share tendencies toward violent or aggressive behavior [23, 24, 36]. Increased frontal lobe activity is often associated with compulsive disorders, such as obsessive–compulsive disorder (OCPD). In contrast, decreased frontal lobe activity may characterize impulsive disorders, such as antisocial personality disorder (ASPD). Third, the biological bases of compulsivity share multiple brain areas and neural circuits with those responsible for language and communication [37]. Therefore, our specific aim was to determine the differences in reading process errors between two distinct groups: one with Antisocial Personality Disorder (ASPD), and another with Obsessive Compulsive Personality Disorder (OCPD). Additionally, we aimed to explore the relationship between these measures and criminal behavior in the prison population.

## Methodology

### Participants

The study participants were recruited from the prison population at the Albolote Penitentiary in Granada, Spain. The participants were selected using stratified probabilistic sampling, and 870 males voluntarily completed the International Personality Disorder Exam (IPDE; [38]). The final study sample consisted of 194 males (mean age = 37.08, SD = 8.81, range = 18 to 55 years), of which 81 (42%) had been diagnosed with ASPD and 113 (58%) with OCPD. The participants were divided into two groups based on the results of the IPDE screening. Group 1 had Antisocial Personality Disorder (ASPD) and included 81 males (mean age = 36.86, SD = 9.32). Group 2 had Obsessive Compulsive Personality Disorder and consisted of 113 males (mean age = 38.78, SD = 8.47). The inclusion criteria were as follows: to be aged between 18 and 55 years old, and to be suffering from ASPD or OCPD. The exclusion criteria were: being aged above 55 years, having a physical illness, being diagnosed with a psychiatric condition (schizophrenia or depression), or currently receiving psychopharmacological treatment.

### Procedure

Members of the prison staff (a psychologist and educator) briefed the participants about the aims of the study. Subsequently, individual interviews were conducted to confirm whether they met the inclusion criteria, and if eligible, they were offered the opportunity to participate in the research. The participants then signed the informed consent form. Once the participants had been briefed, interviewed, and agreed to participate, the prison staff (psychologist and educator) collected the relevant sociodemographic data and administered the International Personality Disorder Examination (IPDE; [38, 39]) and The Symptom Checklist-90-R (SCL-90-R; [40]). Those participants with Antisocial Personality Disorder (ASPD) and Obsessive–Compulsive Personality Disorder (OCPD) were selected. They took part in an individual session in which they completed the measures indicated below. Each participant was assigned a personal number to maintain the anonymity of their identity and data. The personal data, personal number, and personally identifiable information were encoded separately and saved in a secured database. At the beginning of the session, participants were reminded of their rights to withdraw from the study at any time and provided written informed consent if they agreed to participate. The personal number was used to identify the responses of each participant. Participants completed the questionnaire on paper and the research staff collected the responses of each questionnaire. It took participants approximately 3–4 h to complete all study measures. At the end of the session, participants were debriefed and thanked for their participation. All the interviews and interactions with participants were conducted in Spanish. Data collection and subsequent statistical analyses were conducted by the authors of this study (psychologists and a speech-therapist). This research study has been approved by the Ethics Committee of the Junta de Andalucia (ref.:0766-N-21, 4 June 2021). As described previously, all participants provided written informed consent before completing the questionnaire. All participants voluntarily agreed to participate.

### Measures

We employed the following measures:

- *Demographic, Crime, and Institutional Behavior Interview.* This interview was devised specifically for this project and consisted of questions designed to gather socio-demographic data, as well as information regarding the types of crimes committed, and any punishment or prison sentences received according to the Spanish prison regulation law (Royal Decree 1201/1981, 8 May, Articles 107 & 108).
- *International Personality Disorder Exam* (IPDE; [38, 39]). This is a diagnostic instrument based on a semi-structured clinical interview, designed according to DSM-5 criteria [19]. The items consist of open questions, multiple-choice questions, and yes/no questions. The items are classified according to the following six categories: work, self, interpersonal relations, affects, reality check, and impulse control. In addition, the IPDE includes a screening questionnaire that reduces the interview administration time by identifying the personality disorders that the person is unlikely to suffer and excluding further questions regarding these disorders. Completion of the IPDE takes between 60 and 90 min and must be administered by trained and experienced professionals. The reliability and stability indices obtained for the IPDE vary between 0.70 and 0.96 [38]. This instrument is one of the most useful and valid tools for assessing personality disorders for research purposes [38, 39].
- *The Symptom Checklist-90-R* (SCL-90-R; [40]). This is a symptom scale that evaluates the degree of psychological distress a person has experienced in the past week. It consists of 90 items using Likert scales with five response options. The instrument is structured according to nine primary dimensions: Somatizations (SOM), Obsessions and compulsions (OBS), Interpersonal sensitivity (IS), Depression (DEP), Anxiety (ANS), Hostility (HOS), Phobic anxiety (PHO), Paranoid ideation (PAR), and Psychoticism (PSIC). There are seven additional items targeting sleep disorders, eating disorders, death-related thoughts, and feelings of guilt. Three global indices of distress are derived from the scores: An Index of Global Severity (IGS) indicating current levels of perceived distress, Total Positive Symptoms (TPS) indicating the total number of present positive symptoms, and the Index of Symptomatic Distress (ISD) evaluating the response style towards symptoms. Reliability studies show that the nine dimensions reach values close to or greater than α = 0.70 and the concurrent and predictive validity of the inventory and its subscales have been demonstrated using other clinical evaluation instruments, screening scales, psychiatric diagnoses, structured evaluation protocols, or recidivism indicators as criteria [38]. We employed the Spanish adaptation of the inventory [41].
- *Battery for the Assessment of Reading Processes in Secondary and High School—Revised (PROLEC-SE-R; *[42]*).* To assess the main reading processes, the PROLEC-SE-R test was used to evaluate lexical, syntactic, and semantic processes. Moreover, this test analyzes and detects reading difficulties such as dyslexia and hyperlexia. It consists of thirteen tasks [42]. In this study, the six-task screening version was applied, which included Lexical Selection (50 items; participants decide whether or not the presented words are real in a time-limited task according to test instructions); Semantic Categorization (90 items; participants determined whether or not the displayed words were an animal in a time-limited task according to the instructions); Grammatical Structures I (24 items; the aim was to verify which sentences correctly described what the images represented in a time- limited task according to the instructions); Grammatical Judgments (35 items; participants decided whether or not the presented phrases were grammatically correct in a time limited-task according to the test instructions); Expository Comprehension (participants read an expository text and completed ten multiple choice questions with four response alternatives, only one of which was correct; time limited task according to the instructions); Narrative Comprehension (participants read a text and answered 10 multiple-choice items about the content of the text; answers could be identified by consulting the text; and there was no time limit for reading the text and answering the questions). The Cronbach’s alpha reliability index reported for the norm of this test is 0.79.

## Results

### Descriptive statistics

Descriptive statistics are presented in Table 1, which shows the sociodemographic variables and those related to drug abuse, alcoholism history, alcohol and drug treatment, and crimes committed according to group (ASPD and OCPD). To examine the differences between the groups in terms of sociodemographic variables (marital status and educational level), alcohol and drug abuse treatment, and crime, regression analyses were conducted from contingency tables, calculating the chi square statistic for the variables analyzed. The OCPD group contained a higher number of married men than the ASPD group, the number of drug and alcohol abusers was higher in the OCPD group than the ASPD group, whilst the OCPD group had a higher number of participants who had received treatment for quitting drugs and alcohol compared with the ASPD group. There were no significant differences in crimes between the groups (ASPD and OCPD). However, members of the OCPD group had been involved in more cases of gender violence, both as the main crime and as a secondary crime.

**Table 1:** Sociodemographic variables and those related to drug abuse, alcoholism history, alcohol and drug treatment and crimes according to group

	**ASPD (*N* = 81)**	**OCPD (*N* = 113)**	χ^2^
**Marital Status (*N* = 194)**			10,916^**^
Single	41 (50,6%)	48 (42,5%)	
Married	10 (12,3%)	35 (31%)	
Divorced	12 (14,8%)	15 (13,3%)	
Widower	1 (1,2%)	0	
Convive with couple	17 (21%)	15 (13,3%)	
**Educational Level (N)**			1,575
Non-elementary	17 (21%)	16 (14,2%)	
Elementary	33 (40,7%)	51 (45%)	
Secondary	21 (26%)	31 (17,4%)	
High School	8 (10%)	12 (10,6%)	
Degree	2 (2,5%)	3 (2,7%)	
**Crime 1 (N)**			3,417
Against life and integrity	10 (12,3%)	13 (11,5%)	
Against Freedom	4 (5%)	7 (6,2%)	
Against Property; Public estate	46 (56,8%)	52 (46%)	
Against Public Health	8 (10%)	20 (17,7%)	
Gender Violence	13 (16%)	21 (18,6%)	
**Crime 2(N)**			8,416
No crime	19 (23,5%)	34 (30%)	
Against life and integrity	15 (18,5%)	10 (9%)	
Against Freedom	1 (1,2%)	6 (5,3%)	
Against Property; Public estate	34 (42%)	40 (35,4%)	
Against Public Health	10 (12,3%)	16 (14,2%)	
Gender Violence	2 (2,5%)	7 (6,2%)	
**Alcohol and Drug Abuse History (N)**			10,487^**^
No Consume	11 (13,6%)	33 (29%)	
Drug Abuse	37 (45,7%)	42 (37%)	
Alcohol	4 (5%)	12 (10,6%)	
Alcohol and drug abuse	29 (35,8%)	26 (23%)	
**Drug Abuse (N)**			25,370^***^
Never	14 (17,3%)	54 (47,8%)	
Sometimes	33 (40,7%)	39 (34,5%)	
Frequently	22 (27,2%)	9 (8%)	
Always	6 (7,4%)	7 (6,2%)	
Very much	6 (7,4%)	4 (3,5%)	
**Alcohol Abuse (N)**			8,216^*^
Never	22 (27,2%)	37 (32,7%)	
Sometimes	40 (49,4%)	61 (54%)	
Frequently	9 (11%)	9 (8%)	
Always	5 (6,2%)	6 (5,3%)	
Very much	5 (6,2%)	0	
**Alcohol and Drug Treatment History (N)**			9,965^**^
Never	18 (22%)	45 (40%)	
Currently in Prison	34 (42%)	32 (28,3%)	
Throughout life in Prison	21 (26%)	19 (16,8%)	
Outside of Prison	8 (10%)	17 (15%)	

^***^p < .001

^**^p < .01

^*^p < .05

### Psychological distress results

To examine differences in psychological distress between groups, we conducted a Multivariate Analysis of Variance (MANCOVA) for a between-groups unifactorial design using Somatizations (SOM), Obsessions and compulsions (OBS), Interpersonal sensitivity (IS), Depression (DEP), Anxiety (ANS), Hostility (HOS), Phobic anxiety (PHO), Paranoid ideation (PAR), Psychoticism (PSIC), Index of Global Severity (IGS), Total Positive Symptoms (TPS) and Index of Symptomatic Distress (PSD) as dependent variables and group (ASPD and OCPD) as the independent variable. Table 2 clearly shows that the OCPD group obtained lower scores on hostility than the ASPD group.

**Table 2:** Mean, standard deviation, significance level and statistical power of The Symptom Checklist (SCL-90-R)

**SCL-90-R**	**ASPD** Mean (SD)	**OCPD** Mean (SD)	Range of Scores	*F*	η
Total SCL-90	40.34 (2.63)	38.12 (19. 11)	5–87	.596	.003
Total Positive Symptoms	52.83 (23.85)	49.20 (23.01)	5–97	1.141	.006
Index Symptomatic Distress	25.99 (18.87)	27.08 (19.60)	5–85	.151	.001
Somatizations	37.59 (23.98)	40.86 (25.06)	5–97	.831	.004
Obsessions and compulsions	44.69 (21.25)	42. 88 (21.52)	5–95	.339	.002
Interpersonal sensitivity	42.65 (22.45)	42.96 (2.28)	5–87	.010	.000
Depression	41.57 (2.00)	43.54 (19.19)	5–90	.478	.002
Anxiety	40. 03 (2.73)	35.42 (2.10)	5–90	2.418	.012
Hostility	51. 67 (2.26)	37. 34 (14.22)	15–85	33.475^***^	.148
Phobic anxiety	40.18 (15.49)	42. 46 (16.82)	20–90	.918	.005
Paranoid Ideation	55. 34 (17. 60)	54. 33 (18. 18)	5–97	.149	.001
Psychoticism	47. 78 (15. 44)	45. 15 (16. 51)	5–95	1.257	.007

*ns* not significant

^***^p < .001

### PROLEC-SE-R results

To explore differences between groups, we analyzed the reading processes measured using the PROLEC-SE-R. Multivariate Analysis of Covariance (MANCOVA) was conducted using a between-group unifactorial design with educational level as a covariate, group (ASPD and OCPD) as the independent variable, and the PROLEC-SE-R scores (Lexical Selection, Semantic Categorization, Grammatical Structures I, Grammatical Judgments, Expository Comprehension, Narrative Comprehension) as dependent variables. The results revealed statistically significant differences between the groups (Wilks’ Lambda = 0.557, F_6,186_ = 24.704; *p* < 0.001).

Since the MANCOVA revealed statistically significant main effects, univariate ANCOVAs were conducted for each level of the dependent variables (Lexical Selection, Semantic Categorization, Grammar Structures I, Grammaticality Judgments, Expository Comprehension, Narrative Comprehension). The ANCOVAs revealed statistically significant differences in Lexical Selection (F_2,191_ = 23.22; Mce = 34.844; *p* < 0.001), these scores being higher in the ASPD than the OCPD group; in Semantic Categorization (F_2,191_ = 8.22; Mce = 10.71; *p* < 0.001) with the ASPD group obtaining a higher score than the OCPD group; in Grammar Structures I (F_2,191_ = 17.55; Mce = 20.07; *p* < 0.001) with the ASPD group scoring higher than the OCPD group; in Grammatical Judgments (F_2,191_ = 11.09; Mce = 12.167; *p* < 0.001) with the ASPD group obtaining lower scores than the OCPD group; in Expository Comprehension (F_2,191_ = 7.26; Mce = 10.187; *p* < 0.001) with the ASPD group showing higher scores than the OCPD group; and in Narrative Comprehension (F_2,191_ = 9.95; Mce = 7.249; *p* < 0.001) with the ASPD group showing poorer performance than the OCPD group (See Table 3).

**Table 3:** Mean, standard deviation, significance level and statistical power of reading (PROLEC-SE-R) according to group

PROLEC-SE-R	ASPD Mean (SD)	OCPD Mean (SD)	Range of Scores	F	η
Lexical Selection	3.05 (1.33)	2.76 (1.37)	1–5	23.223^***^	.196
Semantic Categorization	2.66 (1.19)	2.34 (1.17)	1–5	8. 222^***^	.079
Grammatical Structures I	2.26 (1.12)	2.25 (1.92)	1–5	17.545^***^	.155
Grammatical Judgments	1.89 (1.14)	1.90 (1.08)	1–5	11.087^***^	.104
Expository Comprehension	2.95 (1.24)	2.81 (1.21)	1–5	7. 256^***^	.071
Narrative Comprehension	3.35 (0.88)	3.50 (0.90)	1–5	9. 952^***^	.094

^***^p < .001

## Discussion

This study set out to explore the differences in reading errors between two distinct groups, one diagnosed with Antisocial Personality Disorder (ASPD) and another with Obsessive Compulsive Personality Disorder (OCPD). An additional aim was to examine the relationship between these measures and criminal behavior within the prison population. Despite the growing interest in developing treatments for incarcerated individuals, more research still needs to be done to improve their speech and language abilities.

Whilst ASPD has been more extensively studied and has been the subject of numerous investigations, OCPD has been relatively underexplored. Individuals with OCPD are more likely to eventually seek treatment due to the debilitating impact of this disorder on their quality of life, stemming from the numerous obsessions and/or compulsions associated with it [43, 44]. Unfortunately, historically OCPD has been mistakenly perceived as a less serious and tangible disorder, and has therefore received somewhat less attention. Consequently, the current version of the DSM-5 [19] highlights the considerable difference between the two disorders in terms of the information that is currently available.

Regarding the sociodemographic differences between these disorders, no significant differences were observed between the two groups. However, it is worth noting that the OCPD group had a higher number of married men than the ASPD group, a difference that could potentially be indicative of a unique profile.

In terms of substance use, participants in the OCPD group consumed less alcohol and drugs than those of the ASPD group. Notably, more individuals in the OCPD group sought treatment to overcome their issues with drugs or alcohol, possibly reflecting the inherent need for control that is characteristic of OCPD.

While no significant differences in crimes were found between the ASPD and OCPD groups, the OCPD group were involved in more cases of gender violence — both as the primary and secondary crime. This observation is particularly striking since the DSM-5 [19] suggests that individuals with OCPD tend to become upset or angry in situations where they cannot maintain control over their physical or interpersonal environment, although their anger is not typically expressed directly.

No significant differences were found between the groups in terms of the measures of psychological distress, except for hostility, where the ASPD group obtained a higher score than the OCPD group. In general, individuals with ASPD show a notable absence of empathy. Additionally, they often display cynicism, cruelty, and a disregard for the feelings and suffering of others, symptoms that are characteristic of this disorder [43, 45].

Our findings indicate differences in reading skills between the ASPD and OCPD groups. First, the results revealed significantly lower rates of Lexical Selection from the PROLEC-SE-R in the OCPD group compared with the ASPD group. This finding suggests that compulsive individuals have greater difficulty in identifying words or pseudowords (with fewer test items answered) or reading new or infrequent words. These difficulties are related to phonological processing, phonological integration, working memory, and the visual system and are congruent with the findings reported by several studies [10, 14, 46] demonstrating that slow reading reflects slow lexical phonological coding. It might therefore be concluded that there is a disruption in the functional aspects of various brain regions, including the supplementary motor area, the premotor cortex, and the putamen. Additionally, alterations could exist in the anterior cingulate nucleus, the cortico-striatum-thalamic-cortical circuits, and the fronto-limbic connections, along with the fascicles of the dorsal or sub-lexical route [12, 14, 20, 47, 48].

Second, significantly lower scores on Semantic Categorization from the PROLEC-SE-R were found in the OCPD group compared with the ASPD group. This observation indicates that compulsive individuals present greater difficulty in the selection of words and pseudowords. These difficulties are associated with semantic categorization, the visual system, phonological and semantic fluency, phonological and syntactic processing, semantic integration and working memory. According to other studies [12, 14, 27, 47–49], this could reflect a disruption in the functional features of the dorsolateral prefrontal cortex, the lateral orbito-frontal cortex, the caudate nucleus, as well as the anterior cingulate nucleus, the cortico-striatum-thalamic-cortical circuits, and the fronto-limbic system.

Third, the results showed that the OCPD group obtained significantly lower scores on Grammar Structures from the PROLEC-SE-R when compared with the ASPD group. This finding suggests that compulsive individuals have greater difficulties in syntax, verbal fluency, generation of phrases and verbs, phonological and semantic processing, word retrieval, semantic integration, working memory, generation and control of language, selective attention, and information processing. At the neural level, these results suggest possible alterations in the dorsolateral prefrontal cortex, the lateral orbito-frontal cortex, and the caudate nucleus, along with the supplementary motor area, the premotor cortex, putamen, the anterior cingulate nucleus, the cortico-striatum-thalamic-cortical circuits, and fronto-limbic connections with the fascicles of the dorsal route (as mentioned previously) and ventral route [12, 14, 27, 49]. The ventral or lexical route is linked to reading processes (involving known and frequent words) and object recognition. Lesions to this route may cause phonetic paraphasia, semantic paraphasia, and the interruption or arrest of language and syntax errors [12, 28, 30].

Fourth, we observed significantly lower scores on Grammatical judgements from the PROLEC-SE-R in the OCPD group compared with the ASPD group. This observation indicates that compulsive individuals present greater difficulties in syntax, verbal fluency, generation of phrases and verbs, phonological and semantic processing, word retrieval, semantic integration, working memory, generation and control of language, selective attention, and information processing. These difficulties could reflect possible alterations in the dorsolateral prefrontal cortex, the lateral orbitofrontal cortex, the caudate nucleus, the supplementary motor area, the premotor cortex, putamen, as well as the anterior cingulate nucleus, the cortico-striatum-thalamic-cortical circuits, fronto-limbic connections, and the fascicles of the lexical and sub-lexical routes [12, 14, 27, 49].

Fifth, significantly lower scores on Expository Comprehension from the PROLEC-SE-R were noted in the OCPD group compared with the ASPD group. This points to the possibility that compulsive individuals present greater difficulties in aspects of reading comprehension, such as syntax, verbal fluency, phonological and semantic processing, semantic integration, working memory, the generation and control of language, selective attention and information processing. This could imply possible alterations in the dorsolateral prefrontal cortex, the lateral orbitofrontal cortex, the caudate nucleus, the supplementary motor area, the premotor cortex, and putamen, along with the anterior cingulate nucleus, the cortico-striatum-thalamic-cortical circuits, the fronto-limbic connections, and the fascicles of lexical and sub-lexical routes [12, 14, 27, 47, 48, 50].

Sixth, we found higher scores on Narrative Comprehension from the PROLEC-SE-R in the OCPD group compared with the ASPD group. This finding could be attributed to the unique characteristics of each type of personality disorder. In particular, individuals with OCPD are not distracted by time constraints and instead focus their efforts on trying to perform tasks correctly, thereby obtaining better results even if they respond to fewer items. In contrast, individuals in the ASPD group could have experienced the effects of fatigue or tiredness when trying to finish quickly and correctly (it is important to note that this task was not timed). These differences could reflect alterations in various brain regions, including the dorsolateral prefrontal cortex, the lateral orbitofrontal cortex, the caudate nucleus, in addition to the supplementary motor area, the premotor cortex and the putamen. Moreover, the anterior cingulate nucleus, the cortico-striatum-thalamic-cortical circuits, and fronto-limbic connections, along with the fascicles of lexical and sub-lexical routes may also be implicated in these findings [12, 14, 27, 49].

Generally, OCPD is characterized by a deficiency in lexical-phonological coding and phonological activation, primarily affecting reading speed rather than accuracy. These results are consistent with those reported by other researchers [9, 14], who have compared different languages and observed similar issues with reading speed in transparent languages, such as Spanish. In contrast, in opaque languages, such as English, the opposite occurs, that is, the problems are related to accuracy rather than speed.

Additionally, our study revealed that individuals with OCPD face challenges in phonological decoding and experience impaired reading comprehension, which are the primary impediments to their reading abilities. These symptoms often resemble those of dyslexia, as reported in a previous study [9] showing that poor word recognition, limited vocabulary, and syntactic difficulties, along with other cognitive and motivational factors, may contribute to the dyslexia-like symptoms observed among the prisoners studied. It is crucial to consider both the acquisition of correct reading speed and the automation of this skill as fundamental elements in the design of interventions aimed at the prison population. Numerous studies [4, 5, 46, 51, 52] have established a clear association between reduced reading speed and deficits in phonological processing. Nonetheless, it is essential that intervention approaches address these aspects separately. Our study has shown that participants with compulsive behavior tend to read more slowly and inaccurately.

The OCPD language deficits found in this study, which have been described by other authors [14, 48] as resembling symptoms of dyslexia, if of significant interest. The chain of events that begins with a reading difficulty and culminates in incarceration carries not only emotional and social consequences for the lives of the prisoners but also imposes an economic burden on the judicial system of each country. According to previous studies [7], it is important to give specialist attention to this school-criminal sequence to alleviate reading problems among the prison population.

We agree with previous authors [16, 51] that advocate for appropriate interventions in correctional facilities to improve reading skills, addressing deficits in decoding or comprehension in people with dyslexia or reading difficulties. Such efforts can also motivate young offenders to continue to improve and broaden their employment prospects. In addition, some studies [14, 15] suggest that young prisoners who participate in intervention programs focused on decoding and linguistic comprehension not only have greater employment opportunities but also show lower rates of recidivism [1, 2]. Thus, a strong correlation exists between participation in correctional education programs and a reduction in criminal recidivism. Consequently, for individuals serving longer sentences, there is a greater need to design vocational-based educational initiatives within prisons.

As with all research, our results should be evaluated in the context of several limitations. For instance, the linguistic disorders studied in the present work require further analysis of the cognitive processes involved in language such as learning, attention, working memory and executive functions. Another limitation is that our sample included only males. This decision was based on three primary reasons: first, we focused on crimes such as gender abuse, which typically involve men as aggressors towards women; second, there were no female inmates serving a prison sentence for partner violence within our study parameters; and third, the prison population contains five times more men than women. Consequently, considering our inclusion and exclusion criteria, conducting this study with women would have been impossible. It would also have been beneficial to compare our groups with control and dyslexia groups. Finally, we did not assess whether our sample (ASPD and OCPD groups) had Attention Deficit and Hyperactivity Disorder (ADHD), dyslexia, or other learning disabilities, which is an issue that should be considered in future research. Nonetheless, this is the first study to examine reading disorders in individuals with compulsive behavior (OCPD) and impulsive behavior (ASPD) in a prison setting. Moreover, this work is among the very few studies that have analyzed performance on the separate components of the PROLEC-SE-R, an aspect that adds value to our research.

The prison population constitutes a significant part of our society, and fully understanding it is not only an act of civic responsibility, but also an ethical imperative in our quest for a more equitable and effective justice system. Studying the prison population is ethically significant for several reasons. First, in a democratic society, transparency and accountability in the justice system are vital. In this regard, examining the prison population allows the broader society to understand how laws and sentences are being applied, and whether they are having the desired impact in terms of rehabilitation and reducing recidivism. Second, focusing on the prison population helps to identify potential inequalities within the justice system, such as the overrepresentation of certain ethnic or socioeconomic groups. By recognizing these problems, we can strive for a more equitable and just system. Finally, if the social objective is to rehabilitate offenders and facilitate their reintegration into society, we must understand their needs and challenges. Research on the prison population offers valuable insights into which programs and approaches are most effective in achieving this goal. In summary, conducting this type of research is not only a moral imperative but a vital step in our pursuit of a justice system that promotes rehabilitation, fairness, and genuine justice within our society. We must approach this issue with seriousness and commitment, respecting the rights and dignity of all individuals involved. Only through such endeavors can we work toward a justice system that embodies these principles.

## Conclusion

OCPD is characterized by slow lexical-phonological coding and phonological activation. Specifically, our OCPD group obtained lower scores on Lexical Selection, Semantic Categorization, Grammatical Structures I, and Expository Comprehension than the ASPD group. However, the OCPD group obtained higher scores on Grammatical Judgments and Narrative Comprehension.

As we have described throughout this work, relatively few studies have analyzed these issues in the prison population with a focus on communication-related aspects and personality traits. Given our society’s overarching objective of promoting the successful reintegration of prisoners into the workforce and reducing rates of criminal recidivism, it is imperative to prioritize these objectives both within and outside the prison. In light of the limited existing research, we believe that it is essential to persist in conducting studies similar to this one. Such research is indispensable for achieving the goal of improving the reintegration of prisoners into the labor market and mitigating the likelihood of repeated criminal offenses. To realize this objective, it is necessary to gain a comprehensive understanding of the factors that impact this prison population, including language and personality disorders.

## Data Availability

The datasets that were generated and analyzed during the study are available from the corresponding author upon request.
